# PPA1 regulates tumor malignant potential and clinical outcome of colon adenocarcinoma through JNK pathways

**DOI:** 10.18632/oncotarget.17381

**Published:** 2017-04-24

**Authors:** Ping Wang, Yi Zhou, Qi Mei, Jing Zhao, Liu Huang, Qiang Fu

**Affiliations:** ^1^ Department of Oncology, Tongji Hospital, Tongji Medical College, Huazhong University of Science and Technology, Wuhan, Hubei, 430030, China; ^2^ Department of Gastrointestinal Surgery, Tongji Hospital, Tongji Medical College, Huazhong University of Science and Technology, Wuhan, Hubei, 430030, China; ^3^ Department of Oncology, Huanggang Central Hospital, Huanggang, Hubei, 438000, China

**Keywords:** colon cancer, JNK, PPA1, prognosis, proliferation

## Abstract

Colorectal cancer (CRC) represents one of the most prevalent malignancies and the third leading cause of cancer death worldwide. Inorganic pyrophosphatase (PPA1) is an enzyme that catalyzes the hydrolysis of pyrophosphate to inorganic phosphate, therefore participates in the energy metabolism. Proteomic studies have demonstrated the up-regulated expression of PPA1 in various tumors, however, its expression pattern in CRC hasn't been reported. In the current study, we used RT-qCR, Western Blot and immunohistochemical (IHC) staining to explore the expression of PPA1 in 113 paired colon cancer tissues and adjacent normal tissues, which revealed that PPA1 was correlated with lymph node metastasis. The prognostic value of PPA1 was confirmed by Kaplan-Meier survival analysis and Cox regression analysis. We further purified PPA1 and obtained the phosphor-JNK1 protein and performed enzymatic studies, which identified that PPA1 can directly dephosphorylate pJNK1, while showed no catalytic activity towards pERK or p-p38 proteins. Moreover, overexpression of PPA1 enhanced cell viability through JNK-p53 signaling pathways, and it may also prevent cell apoptosis by inhibiting Bcl-2 and Caspase-3 cleavage. To our knowledge, this is the first study demonstrated the expression and clinical significance of PPA1 in colon cancer, which also provided evidence that figuring out PPA1 specific inhibitors can be invaluable in the future chemotherapy development towards colon cancer.

## INTRODUCTION

Colorectal cancer (CRC) represents the third most common cancer worldwide with more than 1.3 million new cases annually [1]. Overall survival (OS) of colon cancer largely depends on the disease stages at the time of diagnosis and surgical resection. Approximately 20% of diagnoses are made in the metastatic stage with a 5-year OS less than 15% [2]. However, the clinical outcomes are still not satisfied even for the patients with localized stage, for example, over 20% of stage II colon cancers will develop disease recurrence after resection of the primary tumor [3]. One of the most important reasons for distinct prognosis is the heterogeneous of colon cancer, which contains a series of molecular events including the genetic and epigenetic changes [4]. Therefore, patients’ response to post-operative treatment varies greatly, and numerous studies have attempted to reconcile differential response to chemotherapy based on understanding the molecular characteristics [5].

One interesting point is that all biological changes in tumor development may associated with changes in the energy supplementation and consumption, thus attract us to start with revealing energy dysregulation in colon cancer. The metabolic dysregulation in neoplasm was firstly reported as early as in 1960s, which demonstrated that neoplastic cells favored glycolysis over oxidative phosphorylation [6]. However, only in recent years has it been increasing studied for the therapeutic potential [7].

Inorganic pyrophosphates (PPi) are generated as byproducts of many metabolic reactions, including nucleic acid, amino acid and fatty acid synthesis, as well as cyclic nucleotide activation [8–10]. Dysregulated PPi metabolism has been associated with diseases as reported previously [11–13]. Inorganic pyrophosphatase (PPA1) is thought to play a role in catalyzing the hydrolysis of PPi molecule into inorganic phosphates (Pi). PPA1 exists widely in nature and plays essential roles in many metabolic processes due to its highly exergonic catalytic function [14]. In addition, the hydrolysis of PPi by PPA1 significantly favors cell biosynthesis processes. Extra energy supplementation besides ATP and up-regulated cell biosynthesis metabolism are important characteristics during malignant transformation and development.

Recently, PPA1 was found to be overexpressed in several types of malignancies by proteomic studies, including hepatocarcinoma [15], ovarian cancer [16], breast cancer [17] and lung cancer [18]. Moreover, PPA1 was reported as a negative prognostic marker in gastric cancer [19], although the underlying mechanisms haven't been investigated. In the present study, we analyzed the expression patterns of PPA1 in colon cancer for the first time, and tried to get insights into its functional pathways through enzymatic experiments, gene transfections and biological studies.

## RESULTS

### Patients characteristics and expression of PPA1

This retrospective study included 113 cases, aged from 41 to 78 years. The primary localization of colon cancer included ascending colon (46/113, 40.7%), transverse colon (16/113, 14.2%), descending and sigmoid colon (51/113, 45.1%). Sixty-three (55.8%) patients were diagnosed with positive lymph node metastasis and classified as TNM stage III. None of the patients enrolled occurred distant metastasis at the time of primary surgery. The detailed characteristics of the patients are shown in Table 1.

**Table 1:** Correlation between PPA1 level and clinical features of colon cancer patients

Variables	Patients	PPA1 expression		P value
	(n = 113)	Low (n = 58)	High (n = 55)	
**Gender**				
Female	30	15	15	0.865
Male	83	43	40	
**Age (years)**				
≤ 62	57	33	24	0.159
> 62	56	25	31	
**Serum CEA (ng/mL)**				
≤ 5	83	46	37	0.148
> 5	30	12	18	
**Location**				
Ascending	46	19	27	0.207
Transverse	16	9	7	
Descending/Sigmoid	51	30	21	
**Differentiation**				
Poor	19	10	9	0.719
Moderate	86	45	41	
Well	8	3	5	
**Largest diameter (cm)**				
≤ 5.0	74	40	34	0.424
> 5.0	39	18	21	
**pT**				
T1-T2	48	29	19	0.097
T3-T4	65	29	36	
**pN**				
N0	50	32	18	0.041*
N1	40	18	22	
N2	23	8	15	
**TNM stage**				
Stage I/II	50	29	21	0.206
Stage III	63	29	34	

* indicates statistically significant (P<0.05).

**Figure 1:**
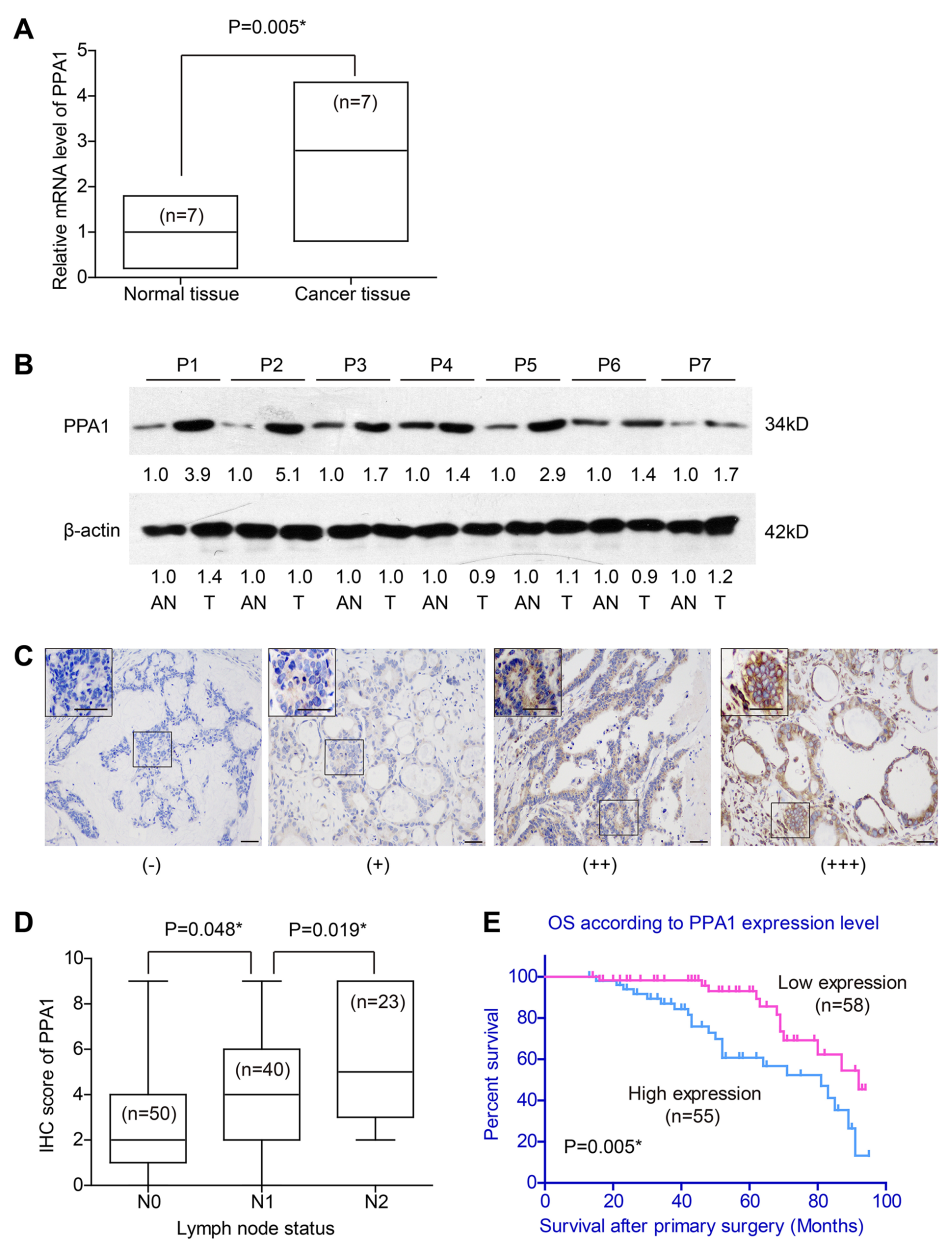
Expression of PPA1 in colon adenocarcinoma tissues (**A**) RT-qPCR results showed higher mRNA levels of PPA1 in cancer tissues than that in adjacent normal tissues (P=0.005). (**B**) Western Blot revealed the different expression levels of PPA1 protein in tumor tissues (T) and adjacent normal tissues (AN). The fold changes were labelled under the bands using AN as control. P1-P7 refer to the patient's number from whom we obtained the fresh-frozen tissues. (**C**) IHC of tumor tissues showed different immunoreactivities. Scale bar: 100μm. (**D**) Patients with advanced lymph node statues exhibited higher PPA1 levels as determined by IHC evaluation. (**E**) High expression of PPA1 indicated poorer overall survival of colon adenocarcinoma patients (P=0.005).

RT-qPCR results showed that PPA1 mRNA level in tumor tissues was higher than that in adjacent normal tissues (P=0.005, Figure 1A). Meanwhile, Western Blot identified the specificity of PPA1 antibody with a single band of the expected size (33kD) in this study, and showed that PPA1 was up-regulated in tumor tissues (Figure 1B). Consistently, IHC results revealed the distinct expression patterns of PPA1 among tumor tissues from different patients, with immunostaining mainly located in cytoplasm (Figure 1C). Low expression of PPA1 was identified in 58 cases (51.3%), while the other 55 cases (48.7%) were classified as PPA1 high expression (Table 1). Moreover, patients with advanced lymph node status showed significantly higher expression of PPA1 (Figure 1D). There was no statistical correlation between PPA1 expression and gender, age, serum CEA, tumor location, tumor differentiation, tumor size or tumor invasion depth (*P*>0.05).

### Prognostic value of PPA1 expression in colon cancer

**Figure 2:**
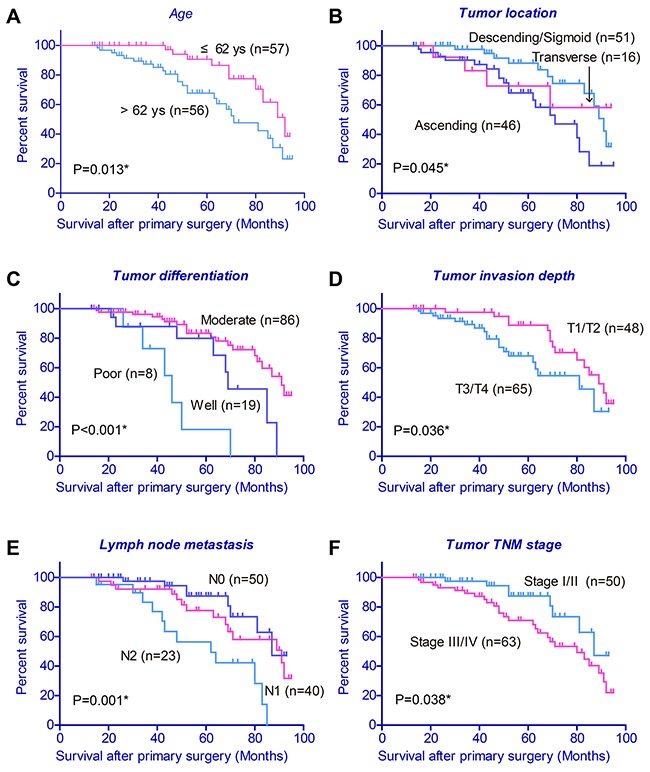
Kaplan-Meier survival curve with respect to clinicopathological characteristics of colon cancer patients Older age (**A**), ascending tumor location (**B**), poor pathological differentiation (**C**), higher T stage (**D**), positive lymph node (**E**) and advanced TNM stage (**F**) were associated with poor clinical outcome.

We investigated whether the OS of colon cancer patients may be related with PPA1 in tumor tissues, as well as with other clinicopathologic factors. Kaplan-Meier analysis revealed that colon cancers expressing high PPA1 levels ((++) or (+++)) correlated with decreased overall survival compared to the low PPA1 group (log-rank test P=0.005, Figure 1E). Notably, the mortality rate of patients in high PPA1 group was higher than that of patients with low PPA1 group (40.0% vs 20.7%, P<0.05) during our follow-up. Univariate analysis also linked poorer prognosis with older age, ascending tumor location, poor pathological differentiation, advanced T stage, positive lymph node and advanced TNM stage (P<0.05, Table 2, Figure 2).

**Table 2:** Kaplan-Meier survival analysis for colon cancer patients

Variable	Cases (n)	5-year OS (%)	Survival months (Mean ± S.D.)	P value
**Gender**				
Female	30	71.7%	76.58 ± 5.05	0.629
Male	83	80.1%	75.04 ± 3.02	
**Age (years)**				
≤ 62	57	90.5%	84.24 ± 2.89	0.013*
> 62	56	63.6%	64.57 ± 3.92	
**Serum CEA (ng/mL)**				
≤ 5	83	79.0%	75.31 ± 2.98	0.950
> 5	30	72.6%	74.35 ± 5.31	
**Location**				
Ascending	46	68.0%	67.53 ± 4.15	0.045*
Transverse	16	72.7%	73.92 ± 8.19	
Descending/Sigmoid	51	88.2%	82.16 ± 3.11	
**Differentiation**				
Well	19	79.9%	69.03 ± 6.22	< 0.001*
Moderate	86	83.3%	80.26 ± 2.79	
Poor	8	18.2%	46.31 ± 5.80	
**Largest diameter (cm)**				
≤ 5.0	74	76.8%	76.39 ± 3.28	0.333
> 5.0	39	79.3%	73.37 ± 4.00	
**pT**				
T1-T2	48	88.8%	81.93 ± 2.93	0.036*
T3-T4	65	68.0%	69.54 ± 3.94	
**pN**				
N0	50	87.4%	81.39 ± 3.39	0.001*
N1	40	77.6%	76.01 ± 4.28	
N2	23	56.3%	59.91 ± 5.77	
**TNM stage**				
Stage I/II	50	87.4%	81.39 ± 3.39	0.038*
Stage III	63	70.9%	70.93 ± 3.58	
**PPA1 expression**				
Low	58	93.0%	82.04 ± 2.93	0.005*
High	55	60.7%	67.90 ± 4.07	

* indicates statistically significant (P<0.05).

Furthermore, we subjected the predicative parameters to Cox multivariate analysis, which identified PPA1 expression as an independent predictor of OS (P=0.038; hazard ratio, 1.391). Other independent prognostic factors included tumor invasion depth, lymph node status and TNM stage (P<0.05, Table 3). Univariate and multivariate analyses verified the clinical significance of PPA1 in predicting clinical outcomes of colon cancer patients.

**Table 3:** Cox-regression analysis for overall survival of colon cancer patients

Variable	HR	95% CI	P value
Age	1.044	0.763 ± 1.471	0.102
Location	1.143	0.947 ± 1.702	0.162
Differentiation	1.374	0.792 ± 2.553	0.125
pT	1.425	1.223 ± 3.029	0.031*
TNM stage^#^	2.348	1.855 ± 4.512	0.015*
PPA1 expression	1.391	1.113 ± 3.106	0.038*

* indicates statistically significant (P<0.05).

### PPA1 regulates cell proliferation capacity

**Figure 3:**
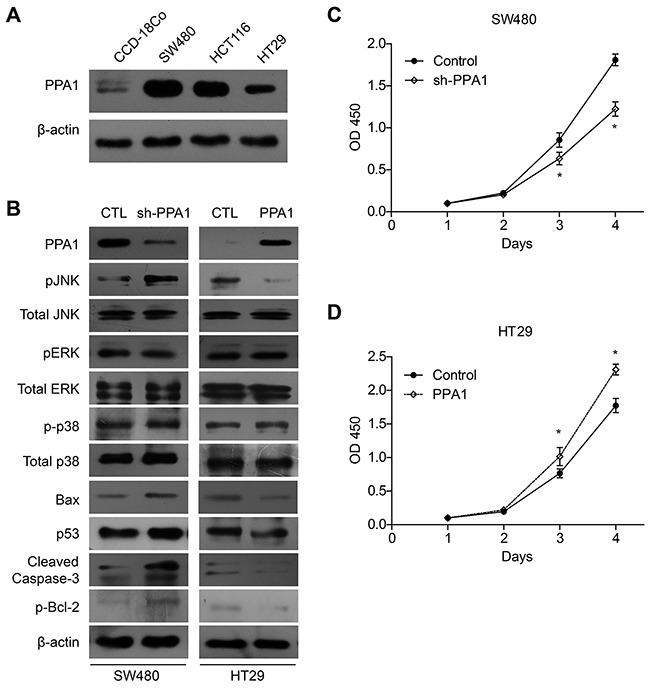
PPA1 promotes proliferation in colon cancer cell lines (**A**) Western Bolt results showed that PPA1 was higher expressed in colon cancer cell lines than that in normal epithelial CCD-18Co cells. SW480 cells showed highest PPA1 expression while HT29 showed lowest PPA1 expression. (**B**) Upon PPA1-silencing, the pJNK level was significantly increased without changes in total JNK protein level. Meanwhile, PPA1 knock-down increased Bax and p53 accumulation, Bcl-2 phosphorylation as well as Caspase-3 cleavage. In contrast, overexpression of PPA1 showed reciprocal regulation towards these signaling proteins. No significant change of pERK or p-p38 levels was observed. Knock-down of PPA1 in SW480 cells inhibited cell proliferation (**C**), while overexpression of PPA1 in HT29 cells showed opposite effect (**D**). Data presented were results from three repeated experiments.

To further elucidate PPA1's relation to colon cancer aggression, we firstly explored its expression in different cell lines. Western Bolt results showed that PPA1 was higher expressed in colon cancer cell lines than that in normal epithelial CCD-18Co cells (Figure 3A). SW480 cells showed highest PPA1 expression while HT29 showed lowest PPA1 expression among the colon cancer cell lines we tested. Therefore, we chose SW480 to perform the silencing of PPA1 with specific shRNA, and overexpressed PPA1 in HT29 cells by plasmid transfection. The efficiency and PPA1 expression in stable cell lines was confirmed by Western Blot (Figure 3B).

Next, we carried out CCK-8 assay to investigate the potential role of PPA1 in regulating proliferation of colon cancer cells. Proliferation curve showed that compared with the control group, cells with PPA1-silencing displayed a significantly impaired proliferation capacity (Figure 3C). On the other hand, PPA1 overexpression in HT29 cells enhanced the cell viability compared with the cells transfected with vector plasmids (Figure 3D, Supplementary Figure 1).

### PPA1 is negatively correlated with pJNK level in colon cancer

The results above demonstrated that overexpression of PPA1 promote proliferation of colon cancer cells and we next want to explore the underlying mechanisms of its functions. Interestingly, we found that under the PPA1-silencing, the pJNK level was significantly increased without changes in total JNK protein level (Figure 2B). However, Immunobolots showed no significantly changes of pERK or p-p38 levels, another two important human MAPKs. Meanwhile, PPA1 knock-down increased Bax and p53 accumulation, Bcl-2 phosphorylation as well as Caspase-3 cleavage. In contrast, overexpression of PPA1 showed reciprocal effects towards these signaling proteins.

**Figure 4:**
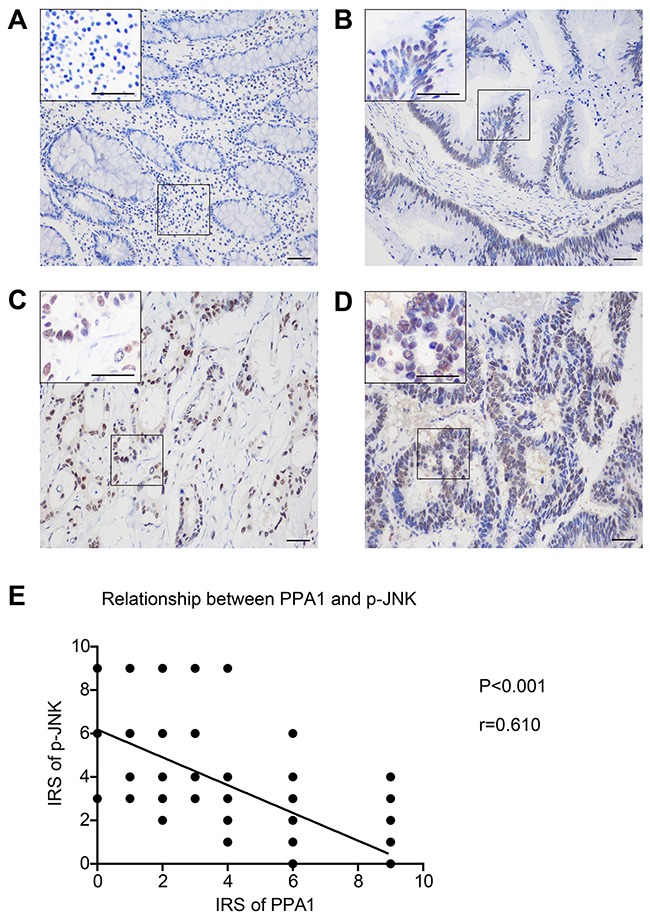
Phosphor-JNK level is negatively correlated with PPA1 expression in colon adenocarcinoma tissues Representative IHC images of pJNK in primary tumor tissues. (**A**) IRS was scored as 0, with negative expression. (**B**) IRS was scored as 2, identified as (+) immunoreactivity. (**C**) IRS was scored as 6, defined as (++) expression. (**D**) IRS was scored as 9, and the staining was classified as (+++). (**E**) Logistic regression curve showed the negative association between PPA1 and pJNK levels (r=0.610, P<0.001).

Results from cellular experiments indicated that PPA1 may negatively regulate pJNK levels and we further performed IHC towards pJNK in colon cancer tissues, which showed nucleus location in cancer cells (Figure 4A-4D). Consistent with Western Blot, the pJNK IHC score was negatively associated with PPA1 expression (Figure 4E).

Taken together, these results implied that PPA1 interacts either directly or indirectly with active JNK (pJNK), and that PPA1 may regulate cellular proliferation in colon cancer cells via the JNK signaling pathway.

### PPA1 directly dephosphorylate pJNK but not pERK or p-p38

**Figure 5:**
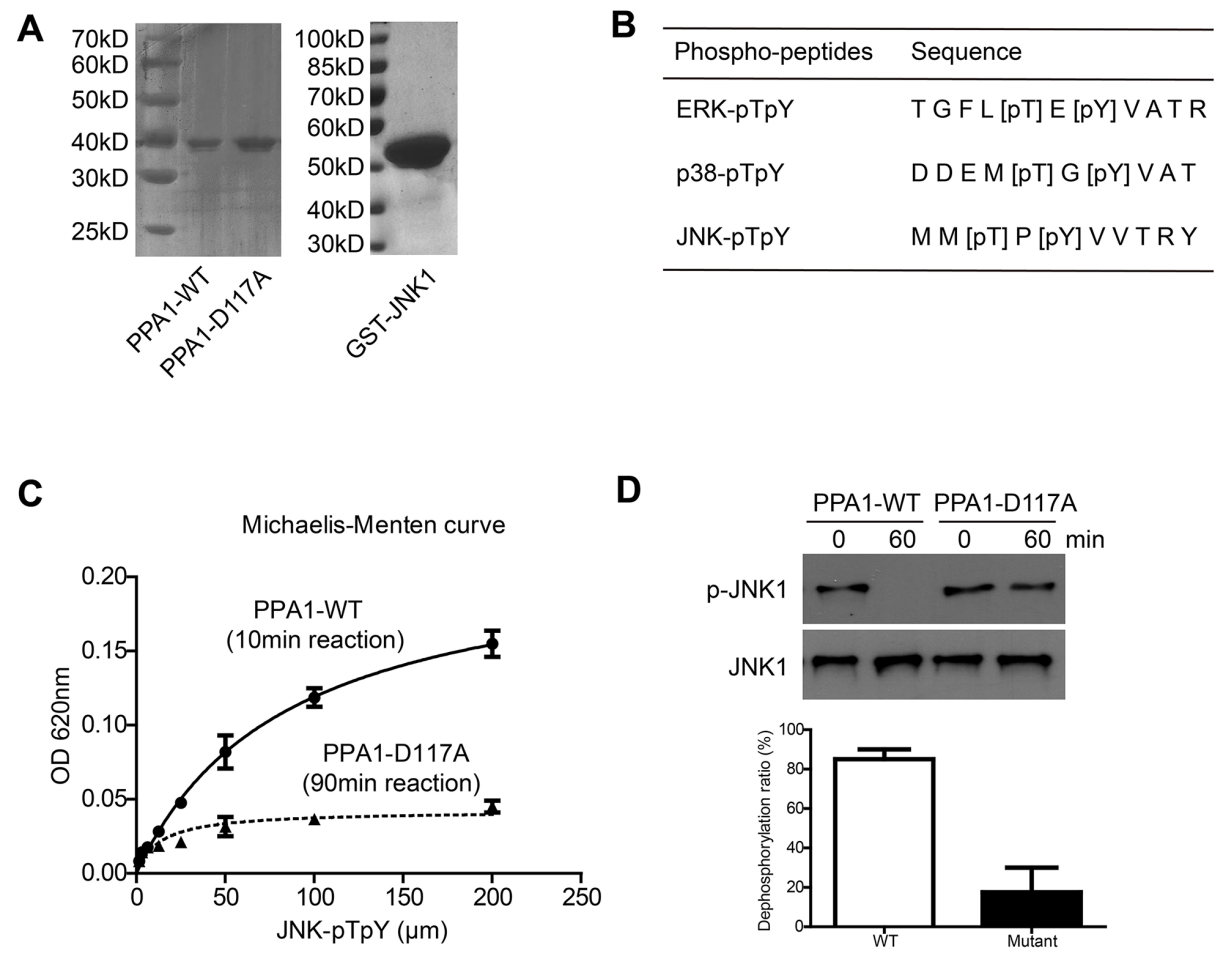
Purification and enzymatic characterization of PPA1 (**A**) SDS-PAGE gels with Coomassie staining showed the high purity of PPA1-WT, PPA1-D117A and JNK1 proteins. (**B**) The synthesized phosphor-peptides and corresponding phosphor-sites were listed. (**C**) PPA1 can dephosphorylate pJNK phosphor-peptides (reaction time was 10min), while its inactive mutant PPA1-D117A (reaction time was 90min) almost abolished its activity. (**D**) PPA1 can directly dephosphorylate pJNK1 protein which was generated by in vitro methods. Data presented were results from three repeated experiments.

We expressed and purified recombinant PPA1 proteins from E. Coli to figure out whether PPA1 was a direct phosphatase towards pJNK. The purity of His-PPA1-WT, His-PPA1-D117A (an inactive PPA1 mutant [20]) and GST-Flag-JNK1 were confirmed by SDS-PAGE (Figure 5A). We then tested the catalytic activity of PPA1 towards different phosphor-peptides (Figure 5B), and found that it almost showed no enzymatic activity on pERK or p-p38 peptides (Supplementary Figure 2). Convincedly, PPA1 can dephosphorylate pJNK on the phosphor-peptide level, whereas the PPA1-D117A mutant showed very slight catalytic activity (Figure 5C).

To better verify that pJNK was a substrate of PPA1, we then generate the pJNK1 proteins through *in vitro* method [21]. After incubation with PPA1-WT for 1 h, the pJNK1 levels was significantly decreased compared to that incubated with PPA1-D117A (Figure 5D). Thus, we concluded that PPA1 can directly bind and dephosphorylate pJNK.

### PPA1 promote cell proliferation through inhibiting JNK activity

**Figure 6:**
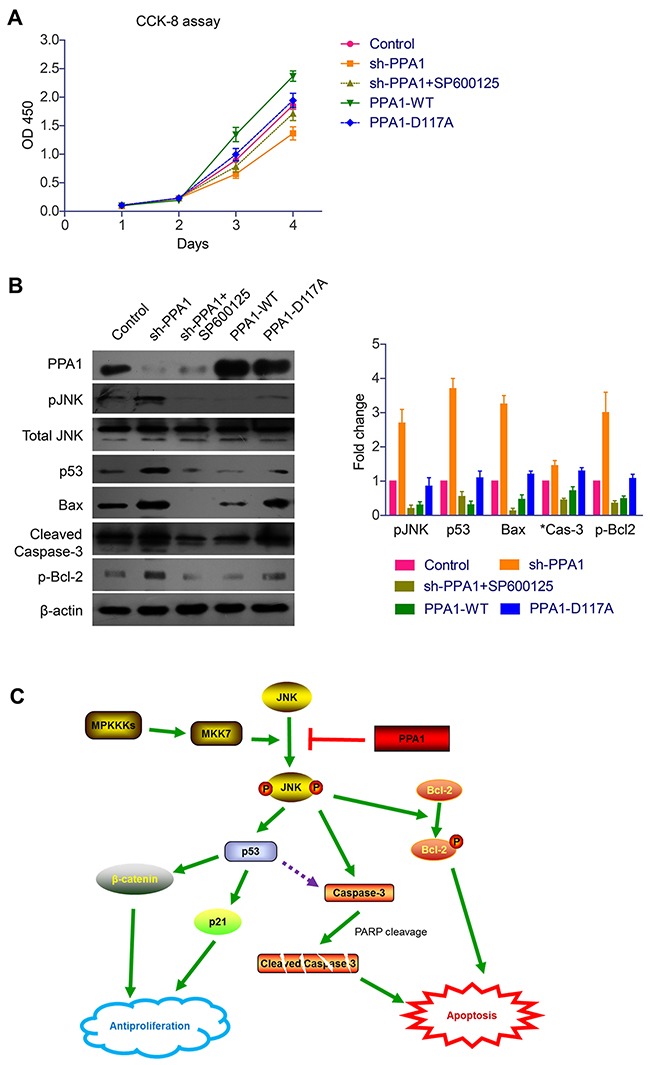
PPA1 enhances cancer cell viability through inhibiting JNK signaling pathways (**A**) CCK-8 assay showed that PPA1 transfection enhanced the cell proliferation of cancer cells, while no significant effect was identified upon PPA1 inactive mutant transfection. In contrast, PPA1-silencing significantly down-regulated the cell proliferation, while JNK inhibitor can impair this antiproliferation effect. (**B**) Western Blot results demonstrated that PPA1 can modulate p53, Bax, p-Bcl-2 and cleaved Caspase-3 levels by recruiting JNK as an intermediate signaling molecular. Histogram showed the quantification results calculated by Image J Software from three experiments. (**C**) Schematically illustrations about the possible molecular and pathways involved in the PPA1 functions towards cancer transformation and development.

Since it has been reported that activated JNK showed antiproliferation effects in cancer cells, we then used JNK specific inhibitor (SP600125) to see whether it can affect the role of PPA1. Cell proliferation assay demonstrated that PPA1-silencing decreased the cell proliferation rate, while this antiproliferation effect was significantly impaired by adding SP600125 (Figure 6A). Meanwhile, SP600125 also eliminated the effects of PPA1-silencing on p53, Bax, cleaved Caspase-3, and p-Bcl2 levels (Figure 6B, Supplementary Figure 1). In addition, PPA1-D117A overexpression showed no significantly effect on either those protein levels or cell proliferation capacity, indicating that PPA1 catalytic activity was essential for its oncogenetic functions in colon cancer cells (Figure 6B, 6C).

## DISCUSSION

CRC incidence is rising due to longer life expectancy and life-style changes, such as poor diet and decreased physical activity [22]. The clinical outcome of localized CRC is far from satisfied [23]. Understanding the basic molecular changes and biological alterations during CRC development and aggression is essential for identifying prognostic markers and developing efficient chemoradiotherapies.

Dysregulated metabolism is now considered as an important hallmark of cancer, and cancer cells are thought to maintain their high proliferation rate through the metabolic alterations [24]. Recently, PPA1 was found to be associated with cell migration, invasion [25] and proliferation [26] of cancer cells, although no clearly mechanism or systematic clinical study was reported. Moreover, the PPA1 expression and its clinical significance in colon cancer haven't been investigated.

In the current study, we identified the up-regulated expression of PPA1 in colon cancer tissues than that in adjacent normal tissues. And PPA1 was demonstrated to be positively correlated with lymph node metastasis, indicating the oncogenetic role of this protein in colon cancer progression. Further, univariate and multivariate analyses revealed that PPA1 was an independent prognostic factor for the OS of colon cancer patients. Overexpression of PPA1 in HT29 cells significantly down-regulated the level of pJNK with little effect on total JNK level. Meanwhile, IHC results identified the negative correlation between pJNK and PPA1 expression levels in colon cancer tissues.

The role of JNK protein in cancer is contradictive. On one hand, mild activation of JNK can promote cell proliferation and invasion predominantly through c-Jun and ATF signaling pathways. On the other hand, strong JNK activation can phosphorylate other substrates such as p53 and Bcl-2, therefore functions as a tumor suppressor [27, 28]. Since consecutively phosphorylation of JNK can definitely induce strong JNK activation, we next explored whether PPA1 can directly or indirectly dephosphorylate pJNK. We carried out protein purification of PPA1, and enzymatic studies showed that PPA1 can directly dephosphorylate pJNK in both phosphor-peptide and phosphor-protein levels, while no catalytic activity towards pERK or p-p38 was detected.

In addition, PPA1 transfection enhanced the cell viability of HT29 cells, while no significant effect was identified upon PPA1 inactive mutant. Thus, PPA1 catalytic activity is required in regulating colon cancer cell proliferation. In contrast, PPA1-silencing significantly down-regulated the cell proliferation, while JNK inhibitor can impair this antiproliferation effects, indicating that PPA1's functions in colon cancer is at least partially through regulating JNK activity. Finally, changes of PPA1 expression can also modulate p-Bcl2 and cleaved Caspase-3 levels, showing the potential role of PPA1 in regulating apoptosis. Consistent with our results, Mishra DR and his coworkers have proved that PPA1-shRNA can significantly decrease the colony forming ability of breast cancer cells [29], which suggest that PPA1 can up-regulate cancer cell proliferation and colony formation. Meanwhile, Luo D. et al showed that PPA1 deficiency induced apoptosis in various cancer cell lines [30]. Therefore, we believe that PPA1 can both regulate the cell apoptosis and proliferation in colon cancer cells.

## MATERIALS AND METHODS

### Patients and clinical specimens

After obtaining approval from the Ethics Committee of Central South University and written informed consent from the patients, fresh colon cancer tissues and pair-matched adjacent normal tissues were obtained from 7 patients between January 2015 and February 2016 at Department of General Surgery, Zhejiang cancer hospital. All fresh specimens from surgery were frozen and stored in liquid nitrogen until required. Another cohort of 113 colon cancer patients was retrospectively enrolled from patients who underwent primary R0 surgical resection from February 2008 to February 2015. Parameters including gender, age, serum CEA (ng/mL), tumor location, pathological differentiation, tumor size (mm), invasion depth (T stage, T1-T4), and status of lymph nodes (N stage, pN0-N2) were reviewed. None of the patients received preoperative treatment, such as radiation or chemotherapy before collecting specimens.

The follow-up was completed on August 31, 2016, with a median patient follow-up time of 57 months (range, 15–96 months). Overall survival (OS) was defined as the interval from the primary surgery to the date of death or the last follow-up. Thirty-four (30.1%) patients passed away by the date of our last follow-up, and the 5-year OS in our cohort is 77.7%

### Antibodies, plasmids and reagents

Anti-PPA1 antibody was purchased from Thermo Fisher Scientific (PA5-22144); pJNK-Thr183/Tyr185 (#4668), total JNK (#9252), pERK-Thr202/Tyr204 (#4370), total ERK (#4695), p-p38-Thr180/Tyr182, p38 (#8690), p-Bcl-2-Ser70 (#2827), p53 (#2524), Bax (#5023) and cleaved Caspase-3 (#9661) antibodies were purchased from Cell Signaling Technologies; anti-β-actin antibody (sc-58673) was purchased from Santa Cruz Biotechnology.

PPA1 cDNA was purchased from Origene (NM_021129) and sub-cloned into pcDNA3.1 vector with puromycin selective gene for generating PPA1 stable cell line. PPA1 prokaryotic expression plasmid were constructed by sub-cloning PPA1 cDNA into pet32a vector containing 6XHis-tag. GST-3XFlag-tagged JNK1a1 plasmid was purchased from Addgene, and D117A mutant was carried out by site-directed mutagenesis using PCR. ShRNA targeting human PPA1 and the scrambled control sequence were synthesized as followed:

PPA1 (5′-GGAATCAGTTGCATGAATATTGGATCCAATATTCATGCAACTGATTCC-3′) [30];

Control (5′-AAAAGCTACACTATCGAGCAATTTTGGATCCAAAATTGCTCGATAGTGTAG C-3′). The JNK specific inhibitors, SP600125, was purchased from Sigma-Aldrich (#S5567).

### RT-qPCR

Total RNA was extracted from fresh-frozen tissues using the Trizol kit according to the manufacturer's protocol. RNA purity and quantification was determined by a Nanodrop1000. RNA was then reversely transcribed to cDNA using SuperScript III reverse transcriptase (Invitrogen, USA). Real time quantitative PCR was performed using a Step One Plus instrument with Taqman® Fast Gen Expression Mastermix (Applied Biosytems, USA). Briefly, PCR cycle parameters were set as: 95°C for 5 min, followed by 40 cycles of 95°C for 20 s, 60°C for 30 s and 72°C for 20 s. The final fluorescence was plotted at the last step of each cycle. The relative mRNA expression levels of PPA1 and GAPDH were further determined by 2^-ΔΔCt^ [31]. Samples were run in triplicate. The primers used for PCR were as followed:

PPA1: 5′-CGCTATGTTGCGAATTTGTTC-3′ (sense primer)

5′-CCAGTATGTTTATCATTGTGCC-3′ (antisense primer);

GAPDH: 5′-GCACCGTCAAGGCTGAGAAC-3′ (sense primer)

5′-ATGGTGGTGAAGACGCCAGT-3′ (antisense primer).

### IHC and IHC evaluation

Paraffin-embedded tissues were cut into 4-μm slides, dewaxed and hydrated, followed by antigen retrieval (in 0.01mol/L citrate buffer solution, pH6.0, heated to boil for 2-3 min in a microwave cooker). Endogenous peroxidase was blocked with a 3% H_2_O_2_ solution. The section was incubated with the blocking goat serum for 15 min, then immunostained with PPA1 or pJNK antibodies (dilution 1:200) at 4°C overnight. Secondary staining was performed with HRP-conjugated IgG and a 3, 5-diaminobenzidine (DAB) peroxidase kit (Beyotime Biotechnology, China). The slides were then counterstained with hematoxylin. Slides were washed then dehydrated in 70% to 100% reagent alcohol and xylenes baths before application of coverslips. The primary antibody was replaced with FBS during the negative control experiments.

Evaluation of IHC results were performed by two independent pathologists, according to both staining intensity (0=negative, 1=weak, 2=moderate, 3=strong) and the number of stained tumor cells (0≤25%, 1=25–50%, 2=51–75%, 3=76–100%). Multiplying intensity with number of stained tumor cells leads to the immunoreactivity score (IRS) which ranges consequently from 0 to 9. IRS with 0-1, 2-3, 4-6, 9 was identified as (-), (+), (++) and (+++), respectively. (-) and (+) staining were grouped as low expression, while (++) and (+++) were grouped as high expression.

### Cell culture and transfection

Three human colorectal cancer cell lines (SW480, HCT116, HT29) and normal colon epithelial cells (CCD-18Co) were used in the present study, all purchased from the American Type Culture Collection (ATCC, USA). All cells were cultured in DMEM (Sigma-Aldrich) containing 10% FBS (Gibco), 100 U/mL of penicillin G, and 100 μg/mL of streptomycin at 37°C in a humidified 5% CO2 atmosphere. Subculture of cells was done every 2 to 3 days for cancer cell lines and 4 to 5 days for CCD-18Co cells with EDTA-containing trypsin. Stable cell lines were generated from cells transfected with pcDNA3.1-PPA1 plasmid or PPA1-shRNA by selecting with puromycin gradient.

### Western blot

Western blot assay was used to detect protein expression and phosphorylation levels. Cell or tissue lysates were subjected to SDS-PAGE, and proteins were transferred to polyvinylidene fluoride (PVDF) membrane (Millipore, USA). The membranes were blocked in Tris-buffered saline (TBS, pH 7.4) containing 5% non-fat milk and 0.1% Tween-20 for 1 h. After that, the PVDF membranes were incubated with the primary antibody at 4°C overnight, followed by the secondary antibody for 1 h at room temperature. The PVDF membranes were then developed using the ECL detection systems.

### Protein purification

The PPA1 wild type (PPA1-WT) and D117A mutant (PPA1-D117A) proteins were expressed in E. coli system and purified using Ni-NTA Purification system (Invitrogen, USA) as instructed by the manufacturer (13). Briefly, guanidine lysis buffer (pH 7.8) was added to bacterial samples, sonicated, and then the cell lysate was centrifuged at 4000 g for 15 min. The supernatant was applied to a column packed with Ni-NTA resin and incubated at room temperature for 30 min with gentle agitation. Afterwards, the column was washed once with denaturing binding buffer (pH 7.8) and then twice with the denaturing wash buffer (pH 6.0). Finally, the resin was washed four times with the native wash buffer (pH 8.0), and proteins was eluted using native elution buffer (pH 8.0). The purified protein was subjected to gel filtration and the final purity was higher than 95% as revealed by SDS-PAGE. The purification of JNK1a1 and subsequent *in vitro* phosphorylation was performed as previously described by others [21].

### Enzymatic analysis

Enzymatic activity of recombinant PPA1 proteins was tested by using phosphor-peptides purchased from China Peptides Co. Ltd (Shanghai, China), which was determined with an inorganic phosphate assay as described by others [32]. Gradient concentrations of phosphor-peptides were incubated with PPA1 in Tris/ acetate buffer (0.05 M Tris, 0.1 M acetate, pH 7.0). 1 μm PPA1 was used as the final concentration for enzymatic analysis, with the phosphor-peptides concentration ranged from 0-200 μm. The reactions were stopped by the addition of BIOMOL GREEN (Enzo Life Sciences), and the absorbance at 620 nm was measured to determine phosphate release. The catalytic data were docked by Michaelis-Menten curve [33].

### Cell viability assay

The proliferation of the cells was determined using a CCK-8 assay (CCK-8, Dojindo, Tokyo, Japan) according to the manufacturer's instruction. Briefly, cells were seeded in 96-well plates at a density of 5×10^3^ cells per well and cultured overnight for cell adhesion. Then with or without stimulation, 10 μL of the CCK-8 reagent was added to each well for 1 hours at designated time points (1, 2, 3, 4 days, respectively). Finally, the absorbance at 450 nm was measured with a Microplate reader. All experiments were performed in triplicate.

### Statistical analysis

The SPSS 15 software package (SPSS, USA) was used for statistical analysis. The association between the PPA1 expression and clinicopathologic features was tested using χ^2^-test. Linear regression model was applied to analysis the relationship between the expression levels of PPA1 and pJNK. The OS rates and mean OS time were assessed by the Kaplan-Meier method and compared with log-rank test. The significance of various survival related variables was assessed by Cox regression model in the multivariate analysis. Comparisons between groups in cell experiments were performed with a two-tailed paired Student's t test. P<0.05 was considered as statistically significant.

## CONCLUSIONS

In all, our study not only demonstrated the expression and predictive value of PPA1 in colon cancer prognosis, but also verified its role as a pJNK phosphatase which highlight its significance as a potential chemotherapy target.

## SUPPLEMENTARY MATERIALS FIGURES


