# Supplementary material for: PPA1 regulates tumor malignant potential and clinical outcome of colon adenocarcinoma through JNK pathways
Source: Oncotarget. 2017 Apr 24;8(35):58611–24. doi: 10.18632/oncotarget.17381 (PMC5601679; doi:10.18632/oncotarget.17381)
Supplement: Supplementary file 1 [file oncotarget-08-58611-s001.pdf]

PPA1 regulates tumor malignant potential and clinical outcome of colon adenocarcinoma through JNK pathways

SUPPLEMENTARY FIGURES

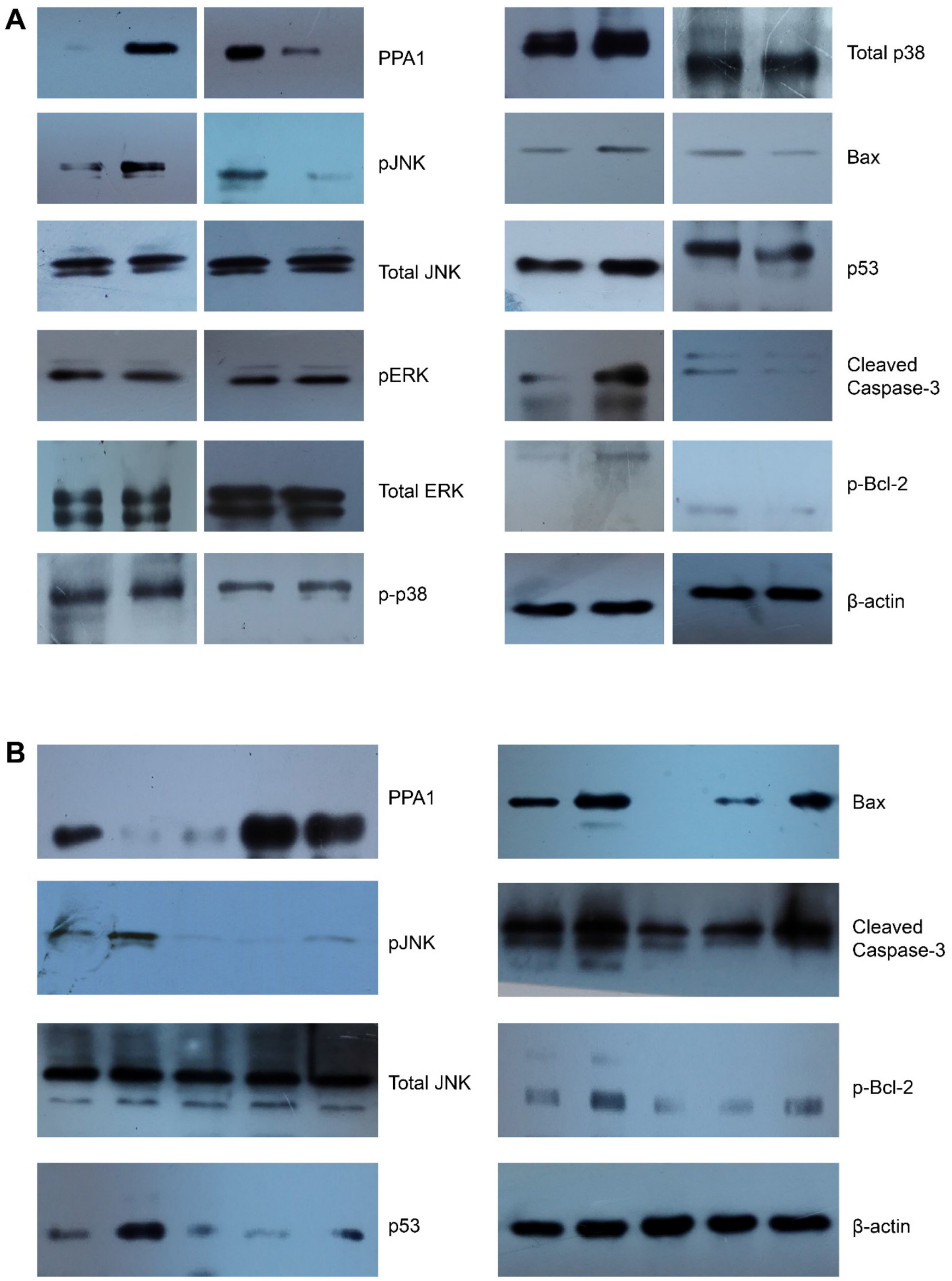

Supplementary Figure 1: (A) Original Western Blot images showed in Figure 3B. (**B**) Original Western Blot images showed in Figure 6B.

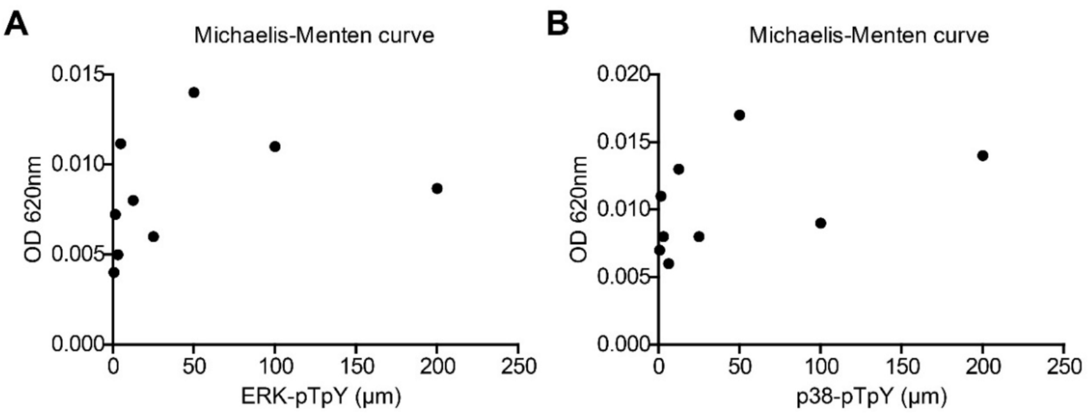

Supplementary Figure 2: Enzymatic analysis of PPA1 to pERK and p-p38 phosphor-peptides. PPA1-WT showed no catalytic activity towards pERK (**A**) or p-p38 (**B**) phosphor-peptides (reaction time was 10min).
